# Arsenic Uptake, Toxicity, Detoxification, and Speciation in Plants: Physiological, Biochemical, and Molecular Aspects

**DOI:** 10.3390/ijerph15010059

**Published:** 2018-01-02

**Authors:** Ghulam Abbas, Behzad Murtaza, Irshad Bibi, Muhammad Shahid, Nabeel Khan Niazi, Muhammad Imran Khan, Muhammad Amjad, Munawar Hussain, Natasha

**Affiliations:** 1 Department of Environmental Sciences, COMSATS Institute of Information Technology, Vehari-61100, Pakistan; g.a92pk@gmail.com (G.A.); behzadmurtaza@ciitvehari.edu.pk (B.M.); drmuhammadamjad@ciitvehari.edu.pk (M.A.); natasha564ag@gmail.com (N.); 2 Institute of Soil and Environmental Sciences, University of Agriculture Faisalabad, Faisalabad 38040, Pakistan; irshad.niazi81@gmail.com (I.B.); khanimran1173@yahoo.com (M.I.K.); munawarhussain452@yahoo.com (M.H.); 3 MARUM and Department of Geosciences, University of Bremen, D-28359 Bremen, Germany; 4 Southern Cross GeoScience, Southern Cross University, Lismore 2480, Australia

**Keywords:** arsenic contamination, bioavailability, potentially toxic elements, oxidative stress, reactive oxygen species, phosphate, plant health

## Abstract

Environmental contamination with arsenic (As) is a global environmental, agricultural and health issue due to the highly toxic and carcinogenic nature of As. Exposure of plants to As, even at very low concentration, can cause many morphological, physiological, and biochemical changes. The recent research on As in the soil-plant system indicates that As toxicity to plants varies with its speciation in plants (e.g., arsenite, As(III); arsenate, As(V)), with the type of plant species, and with other soil factors controlling As accumulation in plants. Various plant species have different mechanisms of As(III) or As(V) uptake, toxicity, and detoxification. This review briefly describes the sources and global extent of As contamination and As speciation in soil. We discuss different mechanisms responsible for As(III) and As(V) uptake, toxicity, and detoxification in plants, at physiological, biochemical, and molecular levels. This review highlights the importance of the As-induced generation of reactive oxygen species (ROS), as well as their damaging impacts on plants at biochemical, genetic, and molecular levels. The role of different enzymatic (superoxide dismutase, catalase, glutathione reductase, and ascorbate peroxidase) and non-enzymatic (salicylic acid, proline, phytochelatins, glutathione, nitric oxide, and phosphorous) substances under As(III/V) stress have been delineated via conceptual models showing As translocation and toxicity pathways in plant species. Significantly, this review addresses the current, albeit partially understood, emerging aspects on (i) As-induced physiological, biochemical, and genotoxic mechanisms and responses in plants and (ii) the roles of different molecules in modulation of As-induced toxicities in plants. We also provide insight on some important research gaps that need to be filled to advance our scientific understanding in this area of research on As in soil-plant systems.

## 1. Introduction

Contamination of air, soil, and water resources with potentially toxic elements (PTEs) is a global environmental issue [1,2]. Most of the PTEs are toxic to plants and animals including humans [3,4,5]. Among the PTEs, arsenic (As) is considered as one of the most highly toxic and carcinogenic [6,7,8,9]. The US Environmental Protection Agency (EPA) and the International Agency for Research on Cancer (IARC) have ranked As and its compounds as a Group 1 human carcinogen [10,11]. The Agency for Toxic Substances and Disease Registry (ATSDR) has ranked As at the top among the 20 priority hazardous substances [12]. It has been reported that one out of every 60 people on this planet is living in a region where the concentration of As in groundwater is 50 µg L^−1^ or greater [2,13].

The ingression of As into the environment, either through natural processes (such as weathering of As-rich minerals in Earth’s critical zone and volcanic activity) or via anthropogenic activities, including the use of wood preservatives, mining, and smelting, excessive use of As-based fertilizers and pesticides in agriculture, and irrigation with As-contaminated groundwater [2,6,14].

The abundance of As is evident due to its presence in more than 200 minerals in the Earth’s crust, of which 60% are in arsenate form, 20% are sulfides and sulfo-salts, and the remaining 20% are in the form of arsenites, arsenides, silicates, oxides, and elemental As [15]. In the soil and water environments, As can exist in four different oxidation states: As(-III), As(0), As(III), or As(V) [16]. Both organic (e.g., monomethyl arsonic acid, arsenobetaine, and arsenosugars) and inorganic species of As are present, the latter being more toxic and mobile than organic As species. Arsenate (As(V)) and arsenite (As(III)) prevail in the natural environment and are highly toxic and mobile; As(III) prevails in reduced conditions, and As(V) is dominant under oxidized environments [2].

Various reactions occurring in soil could affect solution- and solid-phase As speciation in soil and as such could control plant uptake of As and its biogeochemical behavior in the soil-plant system [17,18]. These dynamic soil reactions affecting As biogeochemical behavior, such as oxidation-reduction, sorption-desorption, precipitation/dissolution, volatilization, biological transformations, formation of As-ligand complex and plant species diversity, could be physical, biological, and chemical in nature [19]. It has been stated that the inorganic form of As is more lethal compared to its organic form [20]. Further, amongst its inorganic forms, As(III) is more soluble and 60 times more toxic and mobile than As(V), as the former has been shown to react with sulfhydryl (–SH) groups of proteins and enzymes, culminating inhibition in cellular function and eventually cell death [21,22,23,24,25,26]. However, some recent studies reported that the organic form of As can be more toxic than inorganic As. For example, Duncan et al. [27] reported that dimethylarsenate (DMA), compared to As(V), was more toxic to wheat. They reported that the germination rates of wheat exceeded 80% under As(V) stress but decreased significantly to 20–40% under DMA stress. Similarly, DMA decreased grain yields by 20–50% compared to As(V).

Arsenic is considered non-essential for plants and other organisms [2]. Arsenic uptake by plant species relies on its total concentration and, importantly, on the speciation of As in soil—which is thought to be dependent upon exchangeable (bioavailable) As concentration in soil [17,18,28]. In plants, As mainly enters as an inorganic form, As(III) or As(V) [29] via transporter proteins that is likely governed by As concentration gradient between growth media and plant cells. To our understanding, information about specific transporters for As uptake by plants is lacking [30]. Being analog to each other, both As(V) and phosphate (Pi) use the same transporters to cross the plasma membrane of the root cell [7,16,31].

**Figure 1:**
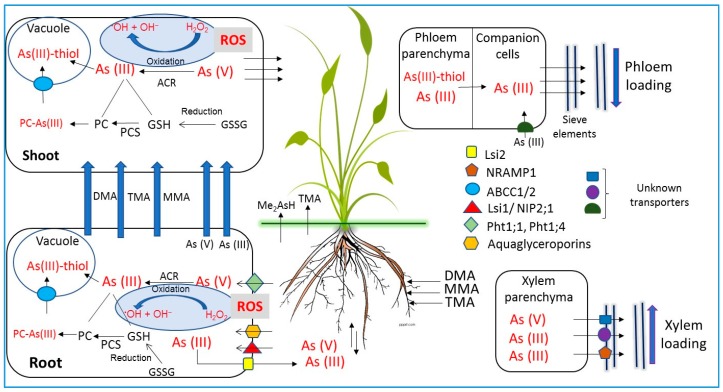
Diagrammatical representation of different components involved in As uptake, transportation, and detoxification in the plants. Modified with permission from Kumar et al. [128].

Inside plants, As can affect growth and productivity due to a plethora of morphological, physiological, biochemical, and molecular alternations (see Figure 1) [6,26,32,33,34]. The most dangerous biochemical effect of As at the subcellular level is the production of the reactive oxygen species (ROS) such as superoxide radical (O^2−^), hydroxyl radical (OH), and hydrogen peroxide (H_2_O_2_) [17,18,35,36]. These ROSs are quite dangerous for plant metabolism and can cause unrepairable damage to important macromolecules, including lipids, proteins, carbohydrate, and DNA [6,37,38,39,40,41,42]. It has been noticed that the generation of the ROS in plants is linked to the conversion of As(V) to As(III) [22,37,43].

Plants protect themselves from oxidative damage caused by the generation of ROSs via increasing activities of the antioxidant enzymes [26,36,40,41,44]. The enzymes involved in the detoxification of ROS include superoxide dismutase (SOD), catalase (CAT), glutathione reductase (GR), and ascorbate peroxidase (APX) [34,41,44,45,46]. Among non-enzyme antioxidants, proline is a well-known osmoprotectant that is largely accumulated in plants under As stress [6,32,47]. It serves as a cell wall plasticizer and helps maintain a minimum level of hydration needed for the normal functioning of cell and protects plants against ROS-mediated damages [22,32,48,49].

The complexation of As with ligands and its vacuolar compartmentation is another mechanism of As detoxification in plants [6,50]. Thiol-reactive cysteine-rich peptides such as phytochelatins (PCs) and metallothioneins strongly bind with As and convert it to a non-toxic form [32,51]. Recent research shows that the exogenous supply of salicylic acid (SA) [26,52], nitric oxide (NO) [26,53], and phosphorous [7,54] can help mitigate As-induced toxicities in plants through various mechanisms.

Considering global significance of As contamination and its impacts on plant growth and biochemistry, it is imperative to critically analyze different relevant published data on As uptake, toxicity, speciation, and detoxification in plants. In this review, we briefly describe the sources and extent of As contamination in soil and water and the speciation of As in soil and discuss the translocation and speciation of As from soil to plants. The physiological, biochemical, and molecular aspects of plants under As stress are also delineated. Arsenic tolerance and detoxification mechanisms in various plant species are addressed in detail. Moreover, the protective role of the exogenous supplies of proline, SA, NO, and Pi for plants under As stress are addressed.

## 2. Arsenic Contamination in Soil and Water Environments

### 2.1. Arsenic Content in Soil

Different soils have varying background concentrations of As depending on the parent material of soils; in most cases, the baseline soil As content can range from 5 to 10 mg kg^−1^ [55]. For European topsoil, an average As concentration of 7 mg kg^−1^ has been reported [56,57]. On the other hand, peats and bog soils are relatively more enriched with As, where average soil As concentrations of up to 13 mg kg^−1^ have been noted. Acid sulfate soils are amongst the other soils that have comparatively more soil As levels [58,59]. For many countries of the world, the United States Environmental Protection Agency (USEPA)’s recommended permissible soil As concentration of 24 mg kg^−1^ has been surpassed due to various anthropogenic activities [60,61].

### 2.2. Arsenic Concentration in Water

Many countries of the world, including Bangladesh, India, China, Pakistan, Chile, Argentina, Mexico, Poland, New Zealand, Canada, Hungary, Taiwan, the United States, and Japan are facing problems of groundwater contamination with As [5,62,63,64,65,66,67]. Approximately 59 districts of West Bengal in India [68], a huge population of Bangladesh, and many areas of Sindh and Punjab provinces in Pakistan are dependent on As-contaminated groundwater for irrigation and drinking purposes [64,65,69,70,71]. More than 3000 μg L^−1^ groundwater As levels have been observed in some countries because As has been released geogenically into aquifers [1,7,65]. According to recommendations of the World Health Organization (WHO), the safety limit of As in drinking water is 10 μg L^−1^, which is being followed throughout the world with exceptions in some South and Southeast Asian countries [9,71,72,73]. Considering the WHO guidelines for As in drinking water, more than 200 million people worldwide are at risk of As poisoning, and about 100 million people in South and Southeast Asia alone face an As-induced risk [74,75,76].

Sea water usually has less than 2 μg L^−1^ of As [77]. Different sources of freshwater, such as lakes, rivers, and streams, have As concentrations ranging from 0.15 to 0.45 μg L^−1^ depending on the sources and geochemical properties of the region [76]. Generally, lake waters have lower As concentrations compared to river waters [78]. Most As-contaminated areas are present around great deltas and the surrounding areas of main rivers that originate from the Himalayan mountain range. The Bengal delta has the highest concentration of As, where more than 88% of 45 million people are affected with high (>50 μg L^−1^) levels of As poisoning [72,76].

## 3. Speciation of Arsenic in Soil

The occurrence of an element in different chemical forms, oxidation states, and mineral phases is known as its speciation [5,79,80,81,82]. The speciation of an element may represent its toxicity and bioavailability in the soil [83,84,85,86]. Nowadays, it is well-known that metal speciation better indicates its biogeochemical behavior compared to total metal content [87,88]. Various species or chemical forms of As in soil include (i) free ionic forms, (ii) precipitated as solids, (iii) adsorbed on soil organic or inorganic constituents, (iv) exchangeable, and (v) structural constituent of primary and secondary minerals [40,89]. The speciation of As is more important than its total concentration regarding its bioavailability and toxicity [1,40,90].

The biogeochemical characteristics of As are not fully dependent on its total concentration in soil [18,91]. For instance, the portion of total As existing as a basic constituent of stable minerals in soil is generally not bioavailable [92]. On the contrary, a portion of total As adsorbed on various organic and inorganic constituents is readily extractable and bioavailable [2,93].

### 3.1. Effect of Soil Chemical Properties on As Speciation and Bioavailability in Soil

The biogeochemical properties of As in the soil-plant system such as mobility, bioavailability, and toxicity greatly depend on its oxidation states [17,18,94]. According to Joseph et al. [95], there are two main oxidation states of As: As(III) and As(V) in nature. The changes in environmental conditions have significant effects on the occurrence of As in diverse oxidation states. The other main species of As in natural environments include monomethylarsonite (MMA(III)), monomethylarsonate (MMA(V)), dimethylarsenite (DMA(III)), dimethylarsinate (DMA(V)), arsenocholine, arsenosugars, arsenobetaine, trimethylarsine oxide, and tetramethylarsonium ions [95].

Inorganic species of As, As(III) and As(V), are present in different forms (e.g., fully protonated As acids or arsenous acid) [96]. The major and thermodynamically stable form of As(V/III) in soil may include HAsO_4_^2−^, H_2_AsO_4_^−^, and H_3_AsO_3_. The presence of As in different inorganic forms (As(III) and As(V)) depends on the pH and redox potential of the surrounding environment. Amongst these, H_2_AsO_4_ is the most frequently occurring species in aerobic soils [97]. Arsenate form of As is very stable and is quickly adsorbed to clay minerals and oxides/hydroxides of Fe/Mn [2]. The arsenite form of As mostly occurs under reduced soil conditions and it is approximately 60 times more soluble, mobile, and toxic than As(V) [2].

Soil pH is considered a master/key variable governing the chemical speciation of metals in soil [98,99,100]. It has been reported that the biogeochemical behavior of As in the soil–plant system mainly depends on soil pH [2]. Under acidic soil conditions (<pH 5.5), As has high mobility and phytoavailability [101]. This is due to the transformation of As into a more soluble As fraction (AsIII) at low soil pH values. Several previous studies reported that change in soil pH affects As speciation in soil and thereby its biogeochemical behavior in the soil system [101,102]. It has been reported that the relative abundance of different inorganic As species (As(III) and As(V) varies with the change in soil pH [102].

Similar to soil pH, soil redox status also affects As speciation in soil. This is because of a well-known fact of high As speciation sensitivity to soil redox conditions [103]. Overall, As solubility of soil increases with as soil redox potential decreases [103]. It is reported that As(V) predominates under oxidizing soil conditions [2,103]. On the other hand, As(V) converts to As(III) under reducing conditions (at low Eh values). In this way, soil redox status can induce a significant effect on As speciation and the geochemical behavior in soil.

### 3.2. The Effect of Soil Microbial Activity on As Speciation and Bioavailability in Soil

In addition to soil chemical properties, soil microbial activities are also known to affect metal bioavailability and speciation in the soil-plant system [84,104,105]. In fact, the microorganisms in soils are involved in numerous important biogeochemical processes that govern the behavior and fate of a metal in the soil-plant system [106]. Soil microbial activity can affect As adsorption/desorption, solubility, bioavailability, mobility, and soil-plant transfer by altering the chemical speciation of As in soil [106,107]. Microorganisms can interconvert As(III) and As(VI) and thus are capable of either solubilizing or immobilizing As in the soil-plant system [2,108]. These microbially induced biotransformations of As play important roles in the biogeochemical behavior of As and is key in risk assessment and remediation studies [109].

Different studies have isolated and reported various species of strict aerobic As(III)-oxidizing and facultative anaerobic As(V)-reducing bacteria from As-contaminated sites [108]. Bacterial species such as *Thermus thermophiles*, *Thermus Aquaticus*, *P. arsenitoxidans*, *Crysiogenes arsenates*, *Bacillus arsenic oselenatis*, *Desulfutomaculum auripigmentu*, *Geospirillum barnesi*, and *Geospirillum arsenophilus* are capable of synthesizing arsenite oxidase and oxidize As(III) into As(V) [110,111]. Similarly, microorganisms can also reduce As(V) into As(III) via dissimilatory reduction. In this process, microorganisms utilize As(V) as a terminal electron acceptor for anaerobic respiration. The bacteria capable of reducing As(V) include *Bacillus arsenic*, *Geospirillum arsenophilus*, *Geospirillum barnesi*, *Crysiogenes arsenatis*, *Sulfurospirillum barnesii*, *Sulfurospirillum arsenophilum*, *Oselenatis*, and *Desulfutomaculum auripigmentu* [112,113].

Some studies also reported an increase or decrease in phytoavalability of As in soil after the inoculation of microbes in soil [2]. This decrease or increase in As phytoavalability is generally attributed to the microbially induced redox transformations of As between As(V) and As(III) [103,107], which differ greatly with respect to their phytoavailability: As(V) is less bioavailable than As(III) because As(V) is more strongly retained by soil constituents than As(III). Stazi et al. [114] reported that soil microorganisms increase As bioavailability by releasing/converting As into its more mobile or water-soluble forms (As III). On the other hand, Hua et al. [115] reported that arbuscular mycorrhizal fungi decreased the phytoavailability of As to the corn plants.

Microbially induced transformations of As from one from to another occurs via different processes/mechanisms such as methylation and demethylation (conversions of inorganic to organic forms and vice versa) [2,104,108,109]. It is reported that microorganisms can biomethylate inorganic As species to organic forms of As [116]. On the other hand, some microorganisms (demethylating) can transform methylated As species to inorganic As forms by biomethylation [117].

## 4. Translocation of Arsenic from Soil to Plant 

It is a general consensus that As is not essential for plants, although the jury is still out on whether or not it is a natural constituent of some plants [2]. According to Gulz et al. [118], a very minute concentration of As in plants could have positive effects in plant species. The concentration of As in plants is usually less than 1.0 mg kg^−1^ dry weight (DW) [119]. Austruy et al. [120] reported an As concentration of <0.1% on a DW basis in different plant species growing on As-contaminated soil. Plants accumulate As in root and transfer to shoot, which can be active (requires energy) or passive (does not require energy) in nature [29].

Normally, plants can take up As in its inorganic form with the help of various transporter proteins (Figure 1) [29], and the main driving force for As uptake is a concentration gradient between source and sink. The mechanism of As uptake by plants varies with the chemical speciation of As. It has been reported that As(V) uses various Pi channels for its entry into the plant cell (Figure 1) [30,121]. This is because P is chemically analogous to As(V). The presence of As(V) in growth medium or P deficiency results in enhanced co-transport of As(V) and Pi [2]. Different Pi transporter proteins (PHT) are the main constituents of P channels involved in As(V) uptake by plants [122,123]. Plants have been reported to contain both high- and low-affinity P transports. The PHT1 proteins are involved in high-affinity transport. On the other hand, the protein involved in low-affinity transport are still unknown. However, some studies reported that some PHT1 proteins may induce low-affinity activity.

On the other hand, plants uptake As(III) via various nodulin-26-like intrinsic proteins (NIPs) (Figure 1). It has been reported that PHT transporters are unidirectional, whereas the NIP transporters are bidirectional. Hence, As(III) can move in both directions between the plant cells and growth medium depending on the difference of As concentration [2]. In plants, As(III) is also reported to use silicon transporters owing to the similarities of As(III) and Si. It has been reported that the expression of influx Si transporter (Lsi1) increases in plants under Si deficiency [124,125]. The accumulation of Si in plants is mainly governed by Lsi1 and efflux Si transporters (Lsi2). The Lsi1 and Lsi2 transporters are localized at proximal and distal sides of epidermal and endodermal cells [125,126], which helps in As transportation across the cells and tissues.

Besides the physico-chemical properties and the chemical speciation of As in growth medium, As uptake by plants is also controlled by various physiological/tolerance mechanisms taking place in different tissues of plant under As stress [2]. In fact, inside plants, As stress can provoke numerous toxic effects at cellular and molecular levels. In order to cope with As toxicity, plants are equipped with various tolerance mechanisms that involve physiological and biochemical changes. These physiological and biochemical changes also affect metal uptake by plants as well as its root-shoot transfer. For example, the reduced metal uptake by plants has been reported as a tolerance mechanism by which plant cells can resist metal toxicity [127]. Thus, the physiological changes inside plants can also affect As uptake by plants.

## 5. Speciation of Arsenic in Plants

### 5.1. Uptake and Transport of Inorganic Arsenic Species

Plants have both high and low affinity for As uptake [129]. Xylem tissues are involved in root to shoot translocation of As and its further distribution among various plant tissues [130] (Figure 1). Arsenate is similar to Pi, and both have competitive uptake by Pi transporters [30,131]. Once inside plant cell, As(V) is reduced to As(III) with the help of As reductase, ACR2 [130,132]. Detoxification of As(III) is accomplished by the formation of its complexes with thiol-rich peptides (Figure 1) [133]. Such complex formation and their further vacuolar storage in root cells is responsible for very low efflux of As(III) and its long-distance transport to other tissues of the plants (Figure 1) [133].

The As hyperaccumulating plant species of Pteris can accumulate As(V), transport more As(III) through xylem, and have less tendency for As(III)-thiol complex formation compared to non-As-hyperaccumulating plant species [130,134,135,136]. According to Logoteta et al. [137], the efflux of As(III) at the root level is regarded as another important mechanism of As detoxification in many plants (Figure 1). Zhao et al. [138] identified a bi-directional As(III) aquaporin transporter (Lsi1), which contributed to approximately 20% efflux of As(III) in rice plants. Hence, it could be inferred that there are some other efflux transporters present in rice. According to Mosa et al. [139], some intrinsic protein (OsPIP) are involved in As(III) uptake by plasma membranes in rice. The speciation of As in phloem is regarded as very crucial for redistribution of As in various tissues within the plant body [140] (Figure 1). However, the role of certain molecules in phloem transport is yet to be explored [130,138].

### 5.2. Uptake and Transport of Organic Arsenic Species

Rice can accumulate methylated As species through their roots using the aquaporin NIP2 (Figure 1) [141]. However, the uptake rate of these methylated organic As species is slower than those of inorganic As species [6]. On the other hand, the translocation of methylated As species from root to shoot via xylem is fast [65,142]. Some organic As species are transported from soil to the xylem stream via the Si influx (Lsi1) and Si efflux transporters (Lsi2) [141,143].

The Lsi1 transporter allows the bidirectional (inclusion and exclusion) movement of Si and As(III) between soil and plant roots cells, whereas the Lsi2 is an efflux transporter and is involved in the exclusion of Si/As(III) into the stele and xylem tissues [2] (Figure 1). Raab et al. [144] conducted a hydroponic experiment using 46 maize species and reported that the transport of DMA(V) from root to shoot was approximately 10 and 3 times greater than MMA(V) and As(V), respectively. Rice grains have been reported to accumulate up to 89% of DMA [145].

## 6. Arsenic Transporters in Plants

In order to understand and manage the harmful effects of As, it is pre-requisite to unravel its transportation in plant tissue [146]. Ma et al. [147] have explored availability of As(III) transporters in *Oryza sativa* L. roots and their participation in As accumulation; they named them NIP transporters (Figure 1). The NIPs belong to one of the four subfamilies of major intrinsic proteins (MIPs) and are commonly known as plant aquaporins.

The transportation of substrates across the aquaporin is regulated by two pore constrictions: (i) asparagine–proline–alanine boxes (which are highly conserved) and (ii) aromatic/arginine (ar/R) [148]. All NIPs have different pore structures at ar/R selective filters. Based on the differences, NIPs have been divided into three subgroups, which includes (i) archetype nodulin-26, which is permeable to glycerol, water, and lactic acid, (ii) a pore size larger than subgroup-I that is permeable to larger solutes like formamide, boric acid, and urea but is less permeable to water [149], and (iii) those responsible for the transport of silicic acid.

The As(III) species is permeable to all NIP subgroups, but its transport is not controlled by an ar/R selectivity filter. As(III) is not permeable to the PIP, TIP, or SIP channel proteins of plant cells [150]. Additionally, As(III) is also transported by silicon influx transporter (Lsi1) and silicon efflux transporter (Lsi2). [16] (Figure 1). A significant fall in As uptake rate was revealed during mutations in Lsi1. Panda et al. [16] further showed that the Lsi2 plays an important function during transport of As(III) from root to shoot and in their accumulation to *Oryza sativa* L. grain.

Arsenate is an analog of Pi, so it shares a similar pathway to cross the plasma membrane of roots via the Pi co-transport system [16,151]. Both As(V) and Pi can compete for a particular group of receptors available on the root cells and, as such, are taken up by the Pi co-transporters of plasma membrane in root epidermal cells (Figure 1) [152]. The above uptake mechanism involves the co-transport of Pi or As(V) and protons, with stoichiometry of at least 2H^+^ for each H_2_PO_4_^−^ or H_2_AsO_4_^−^ [152].

Over 100 Pi transporters have been identified in plants having either a high or low affinity towards Pi molecules. For example, PHT1 proteins are classified as high-affinity transporters, whereas representative proteins for low-affinity transporters have not been identified yet [20]. The transporters, as mentioned above, have been shown to express in root cells of the plants and can accumulate Pi from the external environment [150]. In addition, one more transporter identified for As(V) is dicarboxylate carrier, which is localized in the inner mitochondrial membrane of the plants [153].

In contrast, some studies reported no or non-significant interaction between As(V) and Pi transporters. For example, Christophersen et al. [154] evaluated the effects of Pi on the uptake and toxicity of As(V) in *Hordeum vulgare* and *Medicago truncatula*. These authors reported that the application of Pi to soil did not affect water extractable As and vice versa. Their data also could not find any evidence of variation in uptake of As(V) in *Hordeum vulgare* and *Medicago truncatula* under P supply. It was proposed that a more complex mechanism of interaction may exist between Pi and As(V) uptake and detoxification in plants.

## 7. Physiological Effects of Arsenic on Plants

### 7.1. Effect of Arsenic on Plant Growth

**Figure 2:**
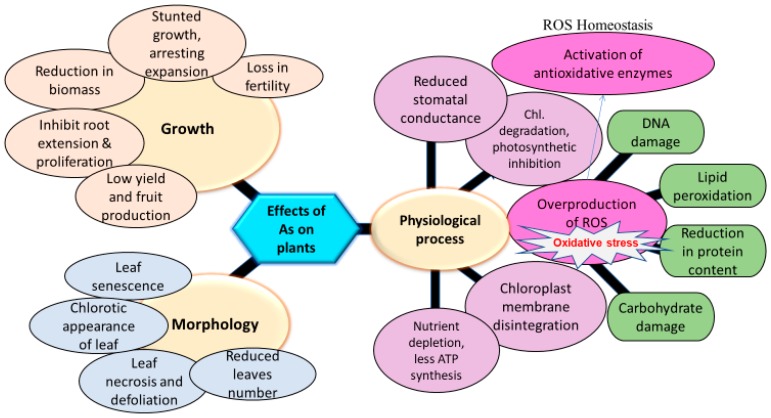
Arsenic toxicity in plants: morphological (reduction in leaf number, chlorosis, necrosis leaf senescence, and defoliation), physiological (reduction in shoot and root growth, restricted stomatal conductance and nutrient uptake, chlorophyll degradation, and limited biomass and yield production), and biochemical (overproduction of reactive oxygen species (ROS), leading to carbohydrate, protein, and DNA damage) responses.

Arsenic availability in soil can disturb normal functioning of plant metabolism, consequently leading to stunted growth and low crop productivity (Table 1, Figure 2) [5,155]. Soil contamination with As in poses a severe threat to human and environmental health [156], thereby affecting thousands of people with As perniciousness throughout the world. Research in the past has indicated that trace amounts of As has a stimulatory effect on plant growth, but high As concentrations are harmful and may begin to outweigh beneficial ones [157].

Imran et al. [13] documented the best performance of *Helianthus annuus* L. seedlings under limited As supply (4 mg/kg soil) but at sufficiently higher concentrations, As hampers critical biochemical and metabolic processes which can result in plant death. Similarly, a significant decrease in plant height has been documented with increasing As load in irrigation water [158]. Under As stress, exposed seedlings of *Cicer arietinum* L. [159] and *Oryza sativa* L. [160] were reported to show remarkable changes in normal functioning of these plants such stunted growth of roots and shoots. Arsenic exposure has also been shown to suppress the number of leaves, leaf area, and fresh and dry mass of plants [24].

Arsenic is reported to disturb biochemical and metabolic pathways such as impeded nutrient absorption, the negative effect on photosynthetic apparatus, the disruption of plant water status, interaction with the functional groups of enzymes and replacement of essential ions from adenosine triphosphate (ATP) in plants growing in As-contaminated soils [2,17,18,161]. Malik et al. [159] also measured a remarkable fall in levels of few important amino acids, like Lys, Met, Pro, Thr, Trp, and Val, in As-stressed *Cicer arietinum* L. seedlings. In addition, As caused wilting, curling and necrosis of leaf blades [20], lower fruit yield, and reductions in leaf area and the rate of photosynthesis [162].

### 7.2. Impact of Arsenic on Photosynthesis of Plants

Arsenic is widely reported to inhibit the rate of photosynthesis in plants (Table 1, Figure 2) [176,177]. After As absorption by plants, light harvesting apparatus can be affected with a reduction in chlorophyll concentrations and photosynthetic activity-II or by suppressing few of the key events of above processes [178]. A remarkable reduction in chlorophyll pigment synthesis was reported due to shortage of the adaptive adjustments of photosystems-I and -II as a result of high As levels. Similarly, a decrease in chlorophyll biosynthesis in *Zea mays* [179], *Trifolium pratense* L. [180], and *Lactuca sativa* [181] seedlings was reported due to As-induced stress.

Toxic effects of As on photosynthetic process could occur either in photochemical or in biochemical steps or even in at both stages. Arsenic caused injuries in chloroplast membrane and disorganized functions of fundamental photosynthetic process [17,182]. Under As exposure, rate of carbon dioxide fixation and functionality of PS-II also reduced considerably [183]. Arsenic has been shown to negatively affect the photochemical efficiency and heat dissipation capacity of a plant, thereby promoting changes in rates of gas exchange and fluorescence emission [6,184]. These results are mainly consistent across studies and suggest that As may cause a decrease in both leaf and root growth, resulting in the appearance of toxicity symptoms such as wilting and violet coloration of leaves [6,185].

### 7.3. Effect of Arsenic on ATP Synthesis

In general, plants accumulate and metabolize As(V) through Pi transport channels. Chemically, As(V) is quite similar to Pi molecules that can disrupt at least phosphate-dependent metabolic aspects during ATP synthesis [186]. As(V) can compete with Pi to bind with transport proteins available on the root surface, but affinity of these transport proteins is quite high towards Pi molecules than that of As(V) [187]. Therefore, increased availability of Pi in rhizosphere may lead to a reduction in As(V) uptake in plants [131,187,188]. However, if the availability of As(V) is greater in soil than that of Pi, As may bind abundantly with root surfaces and transport to the plant cell, thereby resulting in As(V) adducts that are short-lived and unstable. Thus, the formation and rapid autohydrolysis of As(V)-ADP establishes a futile cycle that uncouples oxidative phosphorylation and photophosphorylation, reducing the ability of cells to produce ATP and carry out normal metabolism [187,188].

It has been reported that As(V) toxicity to plants mainly occur via the replacement of Pi in key biochemical processes [20]. Arsenate is able to react with Pi or a Pi-ester as a substrate, thereby disrupting the biochemical process [189]. These As(V)-sensitive processes include RNA/DNA metabolism, phospholipid metabolism, oxidative phosphorylation, glycolysis, and protein phosphorylation/dephosphorylation [20].

An important Pi-requiring reaction in plants is the phosphorylation of ADP to ATP in plastid thylakoid membrane and the mitochondrial inner membrane. However, during this reaction, mitochondrial enzyme may form ADP-As(V) in the presence of As(V) and/or deficiency of Pi [189]. Previously, it has been reported that the phosphorylation of ADP to ATP or the formation of ADP-As(V) are chemically analogue [190]. In fact, some mitochondrial enzymes (F1Fo-type ATP synthase) are capable of recognizing/reacting equally well with Pi and As(V) [191]. In this way, As(V) can disrupt ATP production and the energy status of the cell.

### 7.4. Effect of Arsenic Toxicity on Membrane Integrity

Under As stress conditions, cellular membranes are sensitive sites of damages, and the extent of membrane damage has been reported as a measure of stress tolerance capacity [17,192,193]. Cellular membranes could be damaged due to imbalanced nutrient and water uptake inside plant cells, thus leading to reduced stomatal conductance (Table 1, Figure 2). Deleterious impacts of As on plant transpiration process are probably the outcome of disturbed nutrient uptake and transport of water [194]. Due to As toxicity, the rate of transpiration was reduced dramatically in *Avena sativa* seedlings [195]. In As-stressed *Pteris vittata* L. and *Pteris ensiformis* L. seedlings, Singh et al. [196] observed a significant (*p* ≤ 0.05) decline in cellular membrane stability index at higher doses of As. These authors reported that, after exposure to 267 µM As for 10 days, *Pteris vittata* had a 78.8% membrane stability index (MSI) and *Pteris ensiformis* L. a 22.3% MSI, compared to control [196]. Recently, membrane damage has been reported as being due to an increase in malondialdehyde (MDA), a membrane lipid peroxidation reaction product, along with electrolyte leakage in As-contaminated *Phaseolus aureus* seedlings [197]. The authors further stated that both these processes became more severe under As-stress at 50 µM.

## 8. Biochemical and Molecular Effects of Arsenic on Plants

Generally, As affects any biological system via two routes, i.e., by direct inactivation of key enzymes, either through interaction with sulfhydryl groups or by replacement of mandatory ions from their active sites, or indirectly, through the burst of ROS, thus resulting in a cascade of irreversible injuries in plants [20]. Various metabolic pathways operating in different cellular compartments, such as chloroplast, mitochondria, and peroxisome, can continuously throw ROSs as their byproducts under their normal aerobic metabolism [40,46,198]. These excessively thrown ROSs are widely known to cause non-specific oxidation of proteins, lipids, carbohydrates, enzyme inactivation, membrane leakage, and DNA damages (Table 1; Figure 2) [40,46,85,199,200].

### 8.1. Arsenic-Induced Reactive Oxygen Species (ROS) Generation

**Figure 3:**
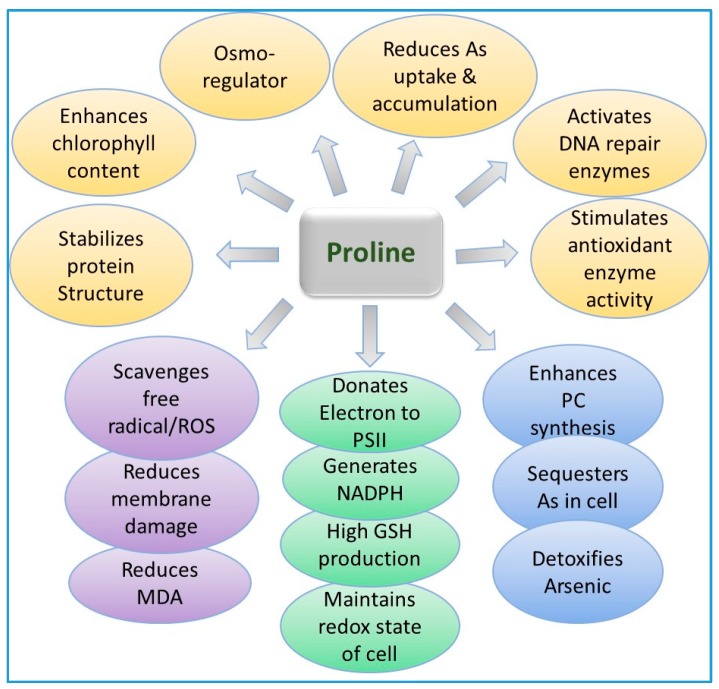
Protective functions of proline in plants under arsenic (As) stress. Proline provides tolerance against As mainly by reducing As uptake, providing osmoregulation, enhancing pigment concentrations, stabilizing macromolecules and cell membranes, maintaining redox state of cell, and scavenging reactive oxygen species (ROS) by through stimulating antioxidant enzymes activities and by vacuolar sequestration of As via enhanced synthesis of PCs.

Under As stress, plants can induce oxidative stress, which causes an imbalance between ROS generation and ROS scavenging [17,18,178,201] (Table 1; Figure 3). The ROS are very unstable oxygen-containing molecules, chemically very reactive, and possess unpaired electron in their valence shell as well as short-lived. Production of ROS is generally toxicity operative due to the presence of PTEs, like As, in chloroplast, mitochondria, and peroxisome as a byproduct of various metabolic pathways functioning within a cell [46,127]. Inside mitochondria, complex-I and -III of electron transport chain (ETC) are prominent locations of Oˉ production [202]. Here, O_2_ is consumed mainly in two pathways: (i) cytochrome oxidase consumes 95% of total O_2_ to produce H_2_O; (ii) in flavoprotein or iron-sulfur center of the NADH dehydrogenase segment, O_2_ is directly reduced to O_2_^−^ [203,204,205].

In ubiquinone cytochrome region of respiratory chain, O_2_ is reduced to O_2_^−^ [204]. Further, during photosynthesis O_2_ is generated in chloroplast which accepts electrons passing through PS-I and PS-II, thereby resulting in formation of O_2_^−^. Under As (or other) stress, only a small portion of total carbon is fixed into CO_2_, resulting in decrease in carbon reduction through Calvin cycle and decline in oxidized NADP^+^ level. This oxidized form of NADP^+^ can serve as the electron acceptor when ferredoxin is over-reduced during photosynthetic electron transfer. Thus, electrons may be transferred from PS-I to O_2_ to form O_2_^−^ by the process called the Mehler reaction [204]. This triggers a chain reaction that generates oxygen radicals quite rapidly [204].

Additionally, O_2_^−^ could be produced by two distinct routes in peroxisomes—either in its matrix where xanthine oxidase catalyzes oxidation of xanthine and hypoxanthine into uric acid and/or in membranes where a small ETC is operative. In its membranes, monodehydroascorbate reductase can participate in O_2_^−^ production [206]. In peroxisomes, H_2_O_2_ is produced by varied processes such as photorespiratory glycolate oxidase reaction, β-oxidation of fatty acids, activity of flavin oxidase, and disproportionation of O_2_^−^ radicals [206].

The increased production of ROS is very dangerous for physiological, biochemical, and metabolic processes taking place inside the plants [46,127,204]. They are largely reported to induce an array of cellular damages at high concentrations and causes changes in redox status of cells, which can serve as a signaling molecule [207]. Thus, effects and/or functioning of ROS would be solely dependent upon its concentration—which tends to increase significantly under stress conditions including As toxicity [37,192,208].

Some studies have demonstrated that enhanced production of ROS in plants is through conversion of As(V) to As(III) [37]. In addition, the leakage of electrons during the reduction of As(V) to As(III) and the inhibition of key enzymes are also important pathways for ROS generation in plants [207].

Conversion of As(V) to As(III) is subsequently followed by a methylation process, which is one of the redox-driven reactions that favors ROS formation [197]. Biomethylation of As generates monomethylarsonic acid, dimethylarsinic acid, trimethylarsonium oxide, tetramethylarsonium ions, arsenocholine, arsenobetaine, and arsenosugars as reported in *Solanum lycopersicum*, *Catharanthus roseus*, and *Agrostis tenuis* [207]. However, methylated forms of As are more reactive with O_2_ and give rise to ROS in the cellular compartments [192]. In addition, methylated forms of As are known to deliver redox-active iron (Fe) species from ferritin within the mitochondria [198]. Afterwards, these active iron species can release harmful O_2_ species via Haber–Weiss reactions [198]. Likely, cytochrome oxidase also catalyzes the conversion of As(V) into As(III), utilizing O_2_ as a final electron acceptor in chloroplast and/or mitochondria, leading towards the production of O_2_^−^.

Li et al. [209] showed high accumulation of ROS in its leaf cells with increasing levels of As in *Triticum aestivum* seedlings. Many other authors have also reported an increase in ROS accumulation in developing embryos of *Zea mays* [210], seedlings of *Triticum aestivum* L. [209,211], *Oryza sativa* [212], *Spinacia oleracea* [213], *Pisum sativum* [17], and *Vicia faba* [18] and seeds of *Anadenanthera peregrina* and *Myracrodruon urundeuva* [128]. According to Kaur et al. [214], approximately 15–28% and 23–82% increases, respectively, in O_2_^−^ and H_2_O_2_ levels were observed after 12 h of exposure to 50 µM of As, which increased further with exposure time and finally reached 104 and 216% after 48 h of exposure.

### 8.2. ROS Homeostasis and Plant Development

The ROS are generally undesirable, highly toxic, and reactive by-products of the aerobic life [215]. Despite their toxic effects (oxidative stress), ROSs have been reported to play key roles in the regulation of cell division and plant development. The essential roles of ROSs in plant development include biotic and abiotic stress responses, hormonal responses, and pollen tube and root-hair growth [216]. ROSs are also involved in signal transduction pathways and the expression of genes [217]. In addition to these essential roles, ROS homeostasis in plants is necessary for signal transduction of programmed cell death, alleviation of seed dormancy, fruit ripening and senescence, resistance against diseases, regulation of cell cycle and stomatal movement, and initiation of mitogen-activated protein kinase cascades [46].

Disturbance in ROS homeostasis has been reported to affect mitosis and cytokinesis in the root-tips of *Arabidopsis thaliana* and *Triticum turgidum* [216,218]. Imbalance in ROS level can induce significant interruption of prophase to prometaphase transition [216]. Some studies have reported the formation of cytokinetic and mitotic aberrations under ROS imbalance. Livanos et al. [216] reported that several root cells of a mutant *Arabidopsis thaliana* (RHD2), which lacks the RHD2/AtRBOHC protein function, exhibited aberrations, compared to those induced by low ROS levels.

### 8.3. Impact of Arsenic on Carbohydrate Metabolism in Plants

The metabolism of basic carbohydrates such as starch and sugars is affected deleteriously in plants under As stress [6], and the accumulation of soluble sugars can occur in response [219]. Jha and Dubey [220] observed a decrease in ratio of reducing and non-reducing sugars in shoots of As supplemented *Oryza sativa*. The conversion of non-reducing sugars, mainly sucrose, into reducing sugars (hexoses) has been observed under the influence of As [220]. It may also reflect suppression of sucrose synthesis relative to available hexose monophosphate.

Additionally, a strong inhibition of the activities of starch degrading enzymes, i.e., α- and β-amylase, and starch phosphorylase has been reported as a result of As-induced plant toxicity [220]. Conversely, an approximately 77–120% increase in starch phosphorylase activity was noticed after As stress to *Oryza sativa* and *Phaseolus aureus* seedlings, resulting in an increased release of soluble sugars [220].

Furthermore, the upregulation in activities of sucrose-hydrolyzing enzymes namely, acid invertase and sucrose synthase, was investigated along with the suppression of activity of sucrose phosphate synthase, under in-situ As toxicity [220]. Acid invertases are the key determinants for production of glucose and fructose (hexoses) from sucrose, and in this regard a direct correlation between its activity and hexose level was calculated by Roitsch et al. [221] and Kaur et al. [214]. The hexoses, synthesized through acid invertase or sucrose synthase, could be later oxidized via the glycolytic pathway [222]. The metabolic impairments as discussed above will lead to altered plant growth and development under the exogenous application of As.

### 8.4. Arsenic Effect on Lipid Metabolism

The oxidation of lipids is considered the most damaging process in plants under As stress [40,64,80,208,223,224]. Cellular membranes could be damaged in response to As stress, leading to increased leakage of cellular electrolytes and many other essential components [193,225]. Additionally, the peroxidation of lipid moieties also takes place in both cellular and organelle membranes when the level of ROS is above a permissible threshold [80,224]. This leads to production of lipid-derived cytotoxic radicals, which affect the normal functioning of a cell or tissue [226].

Significantly, three important steps can be involved in cascade of lipid peroxidation reactions including initiation, progression, and termination [85,193,224]. In cellular membranes, the peroxidation of lipid molecules is initiated by the OH radical [193]. The OH radical abstracts one hydrogen atom from an unsaturated fatty acyl chain of a polyunsaturated fatty acid (PUFA) residue. Under aerobic conditions, O_2_ gets added to a carbon-centered lipid radical of the fatty acid and forms ROO˙. This ROO˙ radical abstracts one H from adjacent PUFA, and peroxidation reaction thus progresses. PUFAs [linoleic acid (18:2) and linolenic acid (18:3)] are very prone to ^1^O_2_ and OH attack and releases mixtures of lipid hydroperoxides as a byproduct [227]. Due to increased peroxidation of PUFA, there is a decrease in membrane fluidity and increases in leakiness, causing severe damage to membrane proteins [228].

Various products of lipid peroxidation could include 4-hydroxy-2-nonenal, malondialdehyde (MDA), hydroxyl and keto fatty acids (Figure 2), and these products can form conjugates with both DNA and proteins [224]. Aldehydes formed in mitochondria are demonstrated to impose cytoplasmic male sterility in *Zea mays* [228]. Recently, scientists have shown a significant increase in MDA in As-mediated lipid peroxidation reactions [37,128,169,177].

Singh et al. [196] observed higher accumulation of MDA in *Pteris ensiformis* than that of *Pteris vittata* and suggested that *Pteris vittata* was able to maintain homeostatic control over photosynthetic light reactions under As stress. This entails that *Pteris vittata* could not produce more ROSs and as such resulted in a low amount of MDA generation. They further noted that increased MDA in *Pteris ensiformis*, due to an impaired cell defense system, was an indication of a precise measure of As-induced oxidative injury [196]. Similarly, in seedlings of *Phaseolus aureus*, an approximately 252% increase in MDA accumulation was observed after 48 h of 50 µM As application [214].

Srivastava et al. [229] have reported an increased generation of MDA (as a thiobarbituric acid reactive substances) in fronds of *Pteris vittata*, *Pteris ensiformis*, and *Nephrolepis exaltata* under high doses of As(V), suggesting that As(V) imposes oxidative stress on these plant species. Likewise, Vázquez et al. [230] reported a high accumulation of both total thiol and MDA in As(V)-stressed *Lupinus albus* plants. The concentrations of thiol and MDA were examined to be high in its nodules than those in leaves, stems, and roots [230]. Further, in roots of *Phaseolus aureus* increased rates of lipid peroxidation were observed with reduced levels of conjugated dienes under As application (50 µM), demonstrating excessive production of ROS and hence oxidative injury [197]. Therefore, the induction of MDA level is closely linked with membrane damage due to the peroxidation of PUFAs, resulting in the generation of the ROS and subsequent oxidative damage [231].

### 8.5. Arsenic Effects on Protein Metabolism

Both As(V) and As(III) could disrupt plant metabolism through distinct mechanism, however, As(III) is 100-fold more toxic than As(V) [232,233]. High toxicity of As(III) is due to its affinity with sulfhydryl groups in proteins, resulting in membrane deterioration and subsequently cell death [234]. Exposure to As caused decreases in total plant protein in *P. ensiformis* and *P. vittata* [196], *Trifolium pratense* [175], *Oryza sativa* [235], *Zea mays* [183], and *Vigna radiata* [236] plant species.

Protein hydrolysis is a vital part, which can contribute varying turnover of individual proteins and free amino acids [233]. Arsenic induced suppression in activities of both nitrate and nitrite reductase have also been held responsible for reduced protein in *Oryza sativa* [235]. Normally, proteins are hydrolyzed into free amino acids and short peptides by actions of the proteases and peptidases [237]. Mascher et al. [175] and Ismail [236] have confirmed a declining trend of proteases in As-exposed *Trifolium pratense* seedlings and *Vigna radiata* cotyledons, which resulted in a low availability of raw materials of structural components, and therefore suppressed growth of developing radicles. Similarly, a decline in protease was documented in As-stressed *Triticum aestivum* [238], *Pteris vittata* and *Pteris ensiformis* [196], *Oryza sativa* [212], and *Phaseolus aureus* [214] seeds.

In addition to hydrolysis, like lipid moieties, proteins are also susceptible to ROS attack [239] (Figure 3). These ROS-induced modifications in proteins may be initiated by leakage of electrons during metal ion-dependent reactions and auto-oxidation of both carbohydrate and lipids [198]. The ROS produced in response to As stress can modify proteins, thereby delivering carbonyls [239]. The amino acids, particularly Arg, His, Lys, Pro, Thr, and Trp, of any protein become oxidized and form free carbonyl groups, which may inhibit or alter the protein activities [240,241]. In turn, protein becomes more susceptible to proteolytic attack [242].

The incidence of protein carbonylation may occur due to the direct oxidation of amino acid side chains (e.g., proline and arginine to γ-glutamyl semialdehyde, threonine to aminoketobutyrate, and lysine to amino adipic semialdehyde) [243]. Proteins with sulfur-containing amino acids and thiol groups are relatively more sensitive toward ROS attack [244]. Cysteine and methionine are very reactive with ROS, especially ^1^O_2_ and OH [244]. Activated oxygen abstracts one H atom from cysteine residues of protein and forms thiol radicals, which could make cross-links with each other and form disulfide bridge [245]. Whilst the methionine residues could be attacked by oxygen and form methionine sulfoxide derivatives [244]. To date, no studies have determined whether or not As-promoted ROS can lead to the induction of oxidation/carbonylation of cellular proteins, particularly in plants, as most research has focused on certain other metals, such as cobalt [246], zinc [247], and copper [248], under salinity stress [208,249].

### 8.6. Arsenic Impact on Changes in DNA Structure

Plant and animal exposure to toxic levels of As has been reported to induce genotoxic responses [170,250]. Many studies have suggested that As genotoxicity is initially linked with the production of ROS during its biotransformation [251]. Thus, the produced ROS can generate DNA–protein adducts and cause DNA and oxidative base damage [252], a break-up of chromatid/chromosome or exchange [250], the formation of apyrimidinic/apurinic sites [253], DNA–protein cross-links [254], chromosomal aberrations, sister chromatid exchange, micronuclei formation, and aneuploidy and deletion (Figure 3) [255].

Another important impact of ROS attack on plant DNA is its base modification, thus releasing 8-oxoguanosine (8-OHdG) from the DNA structure [256]. This 8-OHdG is one of the highly mutagenic miscoding lesions directing G:C to T:A transversion mutations [257]. The accrual of 8-oxoguanine (8-OHdG) adducts, in response to As exposure, has been evaluated in many plant tissues [258,259]. Similarly, single-strand breaks in DNA can be caused directly by ROS or indirectly at time of the base excision repair mechanism [260]. Additionally, As is known to replace P in phosphate-groups of DNA, resulting in organo-arsenical compounds that are toxic and hard to be metabolized by plant system [261,262].

Arsenic is one of the well-known inducers of chromosomal and chromatid aberrations [263] and has also been used to raise the frequency of the micronuclei in peripheral root tip cells of both *Zea mays* and *Vicia faba* [264]. In addition, reductions in telomere length and inhibition in DNA repair processes, such as nucleotide excision repair and base excision repair, are also possible causes of As-induced genotoxic effects in affected plant tissues [253].

It has been reported that the disappearance of normal RAPD bands could be associated with the events of DNA damage, such as point mutation or chromosomal rearrangement induced by genotoxic elements [265,266]. Similarly, Ahmad et al. [170] has shown that the frequency of RAPD band loss increased with rising concentrations and durations of As exposure in *Oryza sativa* seedlings. The changes found in the RAPD profiles of As-affected tissues could be regarded as modifications in genomic template stability, which can be compared with changes in both physiological and biochemical parameters [267,268,269].

## 9. Detoxification Mechanisms of Arsenic in Plants

Plants are able to reduce toxic metal/metalloid, such as As, induced disturbances up to a limited extent by utilizing a number of mechanisms, i.e., by synthesizing metal binding proteins (e.g., metallothioneins and PCs) [32,50,270], and through the elimination of toxic elements from cells by specific transporters or compartmentalization [32,39,51] (Figure 1). In addition, under a wide range of stress conditions, members of the plant defense system (both enzymatic and non-enzymatic) become hyperactive and have better control over the ROS, which is a prime facilitator of cellular injury [43,52,76,271,272,273].

Plant species can also detoxify As-induced toxicity by the production and/or accumulation of compatible solutes or osmolites such as glycinebetaine [274], proline [233], and mannitol [275]. It was reported that the accumulation of osmolyte is an important attribute for protection and survival of plants under stress [276]. Moreover, As-regulated oxidative stress could be successfully reversed in plants by exogenous supply of proline [233,277]. Sodium nitroprusside, a source of NO [39,278,279], SA [52,280], and Pi [7,54], has also been shown to provide tolerance against As in many plants.

### 9.1. Arsenic Complexation and Sequestration in Plants

The sulfhydryl (–SH) groups of peptides such as PCs and GSH have a strong affinity for As(III) [150,281]. It has been reported that As(III) and GSH constitute a (GS)3-arsenite complexation with cysteinyl sulfhydryl group as the As(III) binding site [130,282]. This complex is stable at acidic pH, but is unstable under basic pH. The toxicity of As(III) is manifested by its binding with the –SH groups of proteins. Such a binding results in the disintegration of protein structures and the inactivation of enzymes [171,283].

Inorganic species of As (As(V/III)) have no affinity for thiol groups, but organic As species such as DMA can strongly bind to sulfur-activated GSH [144]. It has been well documented that As detoxification and resultant tolerance against As in non-As-hyperaccumulators can occur via the formation of complexes of As(III) with PCs [130,150] (Figure 1). Different types of PCs are biosynthesized and accumulate in plants when they are exposed to As(III) or As(V) in growth medium [51,171,270,281,284,285].

It has been reported that plant became hypersensitive to As under treatment with l-buthionine-sulfoximine (BSO) (a potential inhibitor of γ-glutamylcysteine synthetase) [283,286]. Ha et al. [287] revealed the evident role of PCs in detoxification of As in *A. thaliana* mutant cad1-3. This mutant is approximately 20 times more sensitive to As(V) than the wild type due to production of a small number of PCs. Both As(III) and As(V) species became more toxic to As-tolerant and non-tolerant *H. lanatus* plants when the synthesis of PCs was repressed by the BSO inhibitor [132,286], indicating an essential role of these PCs in As detoxification.

On the other hand, Hartley-Whitaker et al. [288] found that under the same levels of As(V) stress, the tolerant clones of *H. lanatus* showed a 15–20 times greater production of PCs than that of the sensitive clone, causing a 50% reduction in root length. This suggests that PCs have a crucial role in the adaptations of plants to As stress. It is thought that the formation of arsenite–thiol complexes can take place in cytoplasm from where these complexes are further transported and stored into the vacuole [130,281]. These complexes remain stable under acidic pH (~5.5) conditions of the vacuole [130,150]. The role of PCs in As tolerance was also confirmed in different rice cultivars [289]. Al-Huqail et al. [290] found that gypsum increased As tolerance of chickpea (*Cicer arietinum*) by reducing its uptake and detoxification through complexation with PCs.

### 9.2. Role of Antioxidant Enzymes in Arsenic Detoxification in Plants

Both forms of As can cause disturbance in cell metabolism and redox potential and oxidative damage, leading to the production of ROS [130,291]. Plants protect themselves from the oxidative stress of free radicals by increasing activities of antioxidant enzymes [44,292]. The hyperaccumulator plants can accumulate a significantly higher concentration of As in their bodies, but they have evolved a variety of As tolerance mechanisms against higher levels of free radicals [293,294].

**Figure 4:**
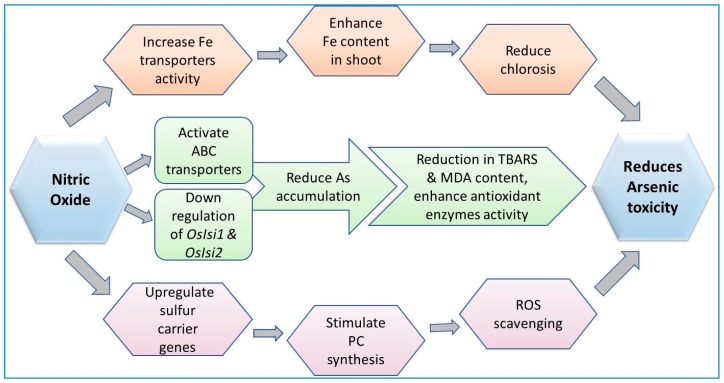
Functions of nitric oxide in plants under arsenic (As) stress. Nitric oxide provides tolerance against As by reducing As uptake through regulating various transporters, reducing chlorosis by increasing iron (Fe) concentrations in shoots, causing vacuolar sequestration of As through enhanced synthesis of PCs, and reducing ROS-mediated oxidative stress by enhanced activities of antioxidant enzymes.

**Figure 5:**
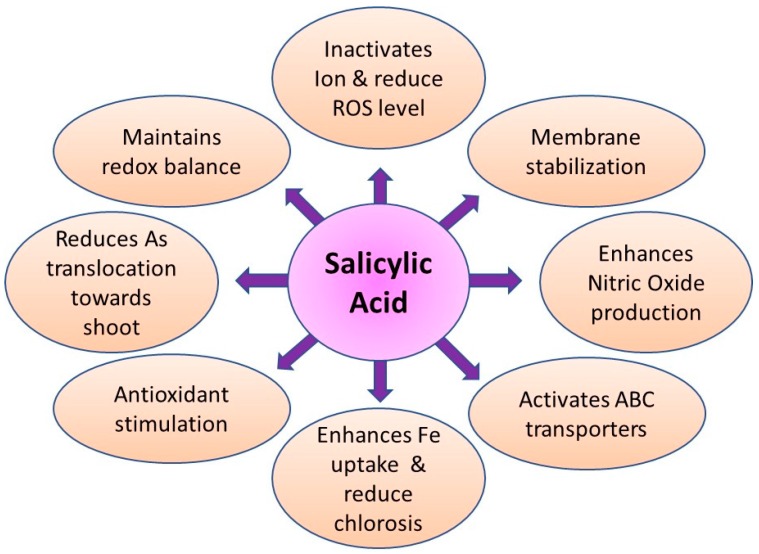
Role of salicylic acid in plants under arsenic (As) stress. Salicylic acid provides tolerance against As by reducing As uptake by regulating transporters, limiting As translocation to shoots, maintaining redox balance of the cell, reducing chlorosis by increasing shoot iron (Fe) concentrations, scavenging reactive oxygen species (ROS), and stabilizing the membrane by enhanced production of NO and antioxidant enzymes.

Upon exposure to As, the enzymes involved in the balancing of these free radicals include superoxide dismutase (SOD), catalase (CAT), glutathione reductase (GR), and ascorbate peroxidase (APX) [44,196,289] (Figure 2, Figure 3, Figure 4 and Figure 5; Table 2). The antioxidative enzymes are generally electron donors and react with ROS to form a neutral and non-toxic end-product. Under biotic or abiotic stress, the activation or the suppression of antioxidant enzymes to scavenge ROS take place in conjugation with each other. Among these antioxidant enzymes, SOD is a key player that constitutes the first line of defense against ROS in plants [295,296]. SOD belongs to a group of metalloenzymes and catalyzes the dismutation of superoxide free radicals (O_2_^−^) into O_2_ and H_2_O_2_ [297]. Arsenic-induced increase in the activity of SOD can be due to an enhanced level of O_2_^−^ or the direct action of As on SOD. In plants, As has been reported to either increase or decrease CAT activity.

SOD has been reported to localize in most of sub-cellular organelles that generate activated oxygen. Its three isoforms, namely, Cu/Zn-SOD, Mn-SOD, and Fe-SOD, have been identified in plants [302]. All three forms are nuclear-encoded and targeted to their respective sub-cellular compartments by an amino-terminal targeting sequence [303]. The Mn-SOD is localized in mitochondria, Fe-SOD in chloroplast, and Cu/Zn-SOD is abundantly in cytosol, chloroplast, peroxisome, and mitochondria [192].

Souri et al. [44] observed a significantly higher increase (116%) in SOD activity in *Isatis cappadocica* Desv, which was exposed to 800 μM As under hydroponic conditions. Such a high increase in the activity of SOD was either due to the effect of As on the SOD gene or due to the overproduction of O_2_^−^ radicals [292,304]. As-induced SOD activities have been observed in many other studies as well [21,26,32,128,273,277,289,305] (see Table 2).

Catalase has been shown to scavenge H_2_O_2_ produced in peroxisomes during photorespiratory oxidation and the β-oxidation of fatty acids [204]. It directly converts H_2_O_2_ into H_2_O without any electron donor [21,44]. Later, authors also reported that CAT reduces H_2_O_2_ irrespective of the level of As in the growth medium and its activity was further increased when As-stressed plants were treated with P probably due to the decrease in lipid peroxidation.

Although there are frequent reports on CAT being present in the cytosol, mitochondria, and chloroplast, the presence of significant CAT activity in these is less established [207].

H_2_O_2_ has been implicated in many stress conditions including As stress [21,34,306]. While cells are stressed for energy and/or rapidly generating H_2_O_2_ through catabolic processes, it is degraded by CAT in an energy-efficient manner [192]. Arsenic stress can cause deviations in CAT activity depending upon the strength, extent, and kind of stress [307]. Accordingly, Souri et al. [44] found that an increase in CAT activity was responsible for detoxification of H_2_O_2_ when the activity of SOD was lower in the shoots of *I. cappadocica*.

Ascorbate peroxidase (APX) is a central component of the ascorbate–glutathione cycle and plays an essential role in the control of intracellular ROS levels [39,130,192]. It is mainly found in cytosol and chloroplasts and considered as an important enzyme in H_2_O_2_ detoxification [308]. APX uses two molecules of ascorbate (AsA) to reduce H_2_O_2_ to water with a concomitant generation of two molecules of monodehydroascorbate. Ascorbate peroxidase is found in organelles which can scavenge H_2_O_2_ produced within it, whereas cytosolic APX abolishes H_2_O_2_ produced in the apoplast or cytoplasm, or that diffused from organelles [309]. Under As stress, the role of APX in mitigating the harmful effects of H_2_O_2_ has been well documented [39,44,52,128,198,289] (see Table 2).

Glutathione reductase (GR) helps plants to maintain a high GSH/GSSG ratio. The GR has been reported to be involved in the NADPH-dependent reduction of oxidized glutathione, and it maintains GSH levels in the cell for proper functioning [32,310]. The oxidative damage caused by As in *I. cappadocica* was partially mitigated by enhancing activities of GR, in order to regenerate GSH [44]. Likewise, the overproduction of GR, under As stress, has been observed in many other plants [32,50,310]. GR has a very preserved disulfide linkage between Cys76 and Cys81. This linkage is broken upon As exposure, and the resultant activity of GR is increased, thus providing defense to plants [44]. It is well-accepted fact that As considerably reduces the GSH level in plants by converting it into PCs. The greater demand for GSH under As-induced oxidative stress conditions is fulfilled by higher GSH turn-over and enhanced GR activities [311].

In fact, plants show tremendous variation in their antioxidant responses during As toxicity [44,130,165,273,289,312] (Table 1 and Table 2). In As-hyperaccumulators, As activates to their antioxidant machinery, resulting in As detoxification and a subsequent accumulation in tissue [229]. In As supplemented *Anadenanthera peregrina* plants, increased activities of SOD, CAT, and APX were investigated initially, which tended to decline later with increasing time of exposure or a high As dose [128]. Many authors [26,52,54,180] have found that, with enhancement in As supply, the activities of antioxidant enzymes were increased in various plant species, such as *Glycine max* L., *Helianthus annuus*, *Triticum aestivum*, and *Oryza sativa*. The As-hyperaccumulating fern species have much higher tolerance against As due to higher activities of antioxidant enzymes and low concentrations of ROS [196,273,294,313].

### 9.3. Role of Proline in Arsenic Detoxification in Plants 

Proline is a well-known osmoprotectant, which is largely accumulated in plants under various stress conditions [22,32,47,314]. It acts as a stabilizer of the cell wall and maintains minimum required hydration level within cells and cell membranes [314,315]. Proline protects plants against ROS-mediated damages [32,48,233] by serving as a singlet oxygen quencher and OH scavenger, so it stabilizes the structure of proteins, DNA, and cellular membranes [22]. It also serves as a transient source of carbon and nitrogen for developing embryos [49,314].

Proline is an important amino acid and is involved in plant growth regulation through signaling processes [316]. Signorelli et al. [317] reported a proline–proline cycle that contributed significantly in scavenging OH radicals. In this cycle, proline first abstracts one H atom and captures an OH ion followed by a second H-abstraction, which also captures another OH and capitulates pyrroline-5-carboxylate. Hence, under the action of pyrroline-5-carboxylate reductase, pyrroline-5-carboxylate is recycled back to proline with concomitant oxidation of NADPH [317]. The enzyme proline dehydrogenase is mainly responsible for the degradation and accumulation of proline in plants [277,318].

It has been reported that metal-tolerant plants over-accumulate proline that enables them to cope-up with water deficit stress by osmotic adjustment [6,289,319]. Thus, proline application effectively regulates to osmotic potential of cells and plays a key role in sustaining plant growth under stresses [49,320,321]. Although not well established yet, proline may also play a key role under the metal-induced disturbance in osmotic potential of cells. When the seedlings of *Triticum aestivum* and *Oryza sativa* were treated with various levels of As(III) and As(V), proline was accumulated in these plants depending on the level of As in growth medium [212,301].

There are a variety of mechanisms through which proline can reduce the toxicity of As: (i) reducing As uptake by changing the structure of the cell wall and protecting plasma membranes [279], (ii) directly quenching As-induced ROS generation [322], (iii) increasing activities of various antioxidants, thus indirectly reducing the damage of ROS [322,323], and (iv) changing the expression of stress-related genes [324] (see Figure 2).

Singh et al. [277] exogenously applied proline to the seedlings of *Solanum melongena* treated with various levels of As(V) under hydroponic conditions. They observed that the application of proline reduced toxicity of As by reducing its uptake by plants. Moreover, better growth of plants under As stress was attributed to an increase in the activities of antioxidants that reduced oxidative damage to seedlings. Proline is involved in the synthesis of PCs, thereby sequestering As and improving the tolerance of plants against As [6,322,325].

It has been reported that chlorophyll concentrations of As-treated plants were increased due to the application of proline [326,327]. Some authors have reported that the endogenous level of proline increased under As stress, which protects plants by reducing As uptake and detoxifying OH radicals [48,277,328]. According to Al-Huqail et al. [290], the application of gypsum increased the As tolerance of *Cicer arietinum*, which was partially attributed to an increase in proline concentrations.

### 9.4. Role of Nitric Oxide in Arsenic Detoxification Processes in Plants 

Nitric oxide (NO), a gaseous free radical, serves as both inter- and intra-cellular signaling agent in plants and can regulate several cellular responses under both normal and stressful conditions [329,330,331]. Sodium nitroprusside is a well-known donor of NO [301,332]. The NO itself is a reactive nitrogen species and has been shown to exert both beneficial and harmful effects depending upon its concentration and localization inside plant cells [306,329,331].

Sub-micromolar concentration of NO has been demonstrated to stimulate plant growth [333], seed germination [236], iron availability [334], and stomatal movement and retard programmed cell death and senescence [279,332,335]. While its millimolar level concentration can afflict plant cells by decreasing the rate of photosynthetic electron transport, leaf expansion, and net photosynthesis and by increasing the viscosity of thylakoid membrane lipids [279,332,335]. Studies have suggested that the exogenous addition of NO can protect plants against As and several other toxic elements [278,331,332]. The nitric oxide application to rice plants growing under As(III) stress significantly reduced root As uptake and its translocation to shoots [26]. Moreover, it mitigated As-induced chlorosis by increasing Fe uptake.

Nitric oxide has also been successfully used to reduce both growth inhibition and ROS-imposed injuries under As stress in *Oryza sativa* [38,53,235], *Festuca arundinacea* [173], *Vigna radiata* [236], *Vicia faba* [336], and *Phaseolus vulgaris* L. [37]. Acting as an antioxidant, the pretreatment of plants with NO has been reported to scavenge ROS completely and enhances tolerance against As [53,227,301,331].

Nitric oxide has been shown to confer protective functions through specific mechanisms: (i) NO reacts with OH radicals to form HNO_2_, thus protecting cells against dangerous OH radical by averting Fenton reaction, (ii) it reacts with lipid radicals such as lipid alkoxy (LO^●^) and lipid peroxyl (LOO^●^), and as such prevents radical-mediated lipid peroxidation, (iii) it reacts with superoxide radical and forms peroxynitrite (ONOO^−^), which have very less toxicity and lifespan than highly toxic H_2_O_2_ and thus stabilizes to other cellular processes, (iv) NO activates antioxidants like SOD, CAT, APX, POX, and (v) it functions as a signaling molecule in the cascade of events leading to changes in gene expression [39,331,337,338,339,340] (see Table 2 and Figure 4).

Due to signal transduction, NO increases activities of antioxidants and stimulates the entire biosynthetic pathway of PCs in roots, which help increase the uptake of sulfate together with synthesis of both amino acids and non-protein thiols [332]. The application of NO boosts As tolerance in *Luffa acutangula* by enhancing cell wall thickness in root epidermis, thereby controlling As absorption and accumulation [279]. Similarly, NO improved the antioxidative capacity, but it reduced auxin degradation and increased ion absorption in stressed plants [341]. Hasanuzzaman and Fujita [301] observed that the application of NO increased relative water concentrations, chlorophyll concentrations, and antioxidant enzymes and glyoxalase activities in wheat seedling under As stress. The exogenous application of sodium nitroprusside to *Vicia faba* seedlings was found to increase seed yield, phytohormones, photosynthetic pigments, and mineral concentrations, thereby reducing As toxicity [336]. Likewise, a 100 µM sodium nitroprusside application reduced the translocation of As from root to shoot in watercress tissues. Moreover, it increased the concentrations of proline, protein, and various antioxidants [278]. In addition to the usual oxidative stress tolerance mechanisms mediated through various antioxidant enzymes, NO has been found to reduce As toxicity by increasing expression of alternative oxidase (Aox1) enzyme in barley (*Hordeum vulgare* L.) [342].

### 9.5. Role of Salicylic Acid in Arsenic Detoxification 

Salicylic acid is a phenolic substance and endogenous plant growth regulator, which participates in physiological processes, such as photosynthesis, growth, nitrate metabolism, flowering and ethylene production [343,344,345]. SA not only plays a crucial role in ascertaining and signaling of defense responses against pathogenic infections but also contributes to several other adverse conditions [346] (Figure 5).

SA can also be involved in abiotic stress signaling, together with plant responses to toxic elements such as As [345]. Singh et al. [26] found that exogenous application of SA to rice plants under As stress was very promising in reducing As toxicity by increasing the endogenous concentrations of both SA and NO. The increased concentration of NO was attributed to the enhanced activities of nitrate reductase. Moreover, the downregulation of the *OsLsi*2 gene, under SA and NO supply, was responsible for reduced As uptake. SA also helps in regulating various cellular activities of plants and takes part in the control of their growth and productivity [343,347]. After application, SA efficiently removed disruptions caused by As in *Arabidopsis thaliana* seedlings [280]. A considerable rise in metal-imposed oxidative injuries and growth inhibition has been observed in *Brassica napus* L. [306], *Oryza sativa* [26,316], and *Glycine max* L. [348] seedlings, which can be successfully eliminated by the exogenous supply of SA.

Salicylic acid has been found to increase the activities of SOD and APX enzymes in plants and provide them with tolerance against oxidative damage caused by metals [324,349,350]. Exogenous application of SA was found to be closely associated with an increased accumulation of proline in plants, thereby alleviating the toxic effects of As and metals [351]. In some stressed plants, the addition of SA was shown to prevent the lowering of both indole acetic acid and cytokinin levels, leading to improved cell division in root apical meristem [347]. Likewise, the application of SA also increases the synthesis of pigments in green plants, resulting in an enhancement of photosynthetic performance and vigorous plant growth [26,348]. Zengin [352] found that the treatment of wheat seedlings with 1 mM SA reduced As toxicity by increasing chlorophyll and protein concentrations and by reducing lipid peroxidation.

Salicylic acid mitigated As-induced toxicity in *Glycine max* L. by reducing production of the ROS and increasing activities of antioxidant enzymes and proline [22]. Singh et al. [38,277] showed that the co-application of SA, compared to pre-application, was more effective in reducing As uptake to *Oryza sativa* L. They reported that SA reduced As(V)-induced toxicity in rice plants by enhancing activities of both enzymatic and non-enzymatic antioxidants and increased concentrations of Asc, GSH, and PCs in plants under As stress.

The application of SA was helpful in mitigating chlorosis by enhancing Fe uptake. Likewise, Saidi et al. [52] reported that SA reduced the adverse effects of As on growth of *Helianthus annuus* L. seedling by decreasing the oxidative stress accompanied by higher activities of ascorbate peroxidase (APX), catalase (CAT), and glutathione peroxidase (GPX). Figure 5 shows the role of SA in As tolerance through various mechanisms.

### 9.6. Effect of Phosphate (Pi) on Arsenic Toxicity and Detoxification in Plants

Many studies have shown that the addition of Pi to plants under As stress has positive effects on plant growth and as such could increase plant tolerance against As stress [7,8,30,128,298]. It is well established that both As and Pi have similar chemical properties and use the same carrier molecules for the uptake in plant roots via plasma membranes [6,234,345,353]. Many genetic studies and physiological data from different species have revealed that As(V) and Pi are taken up by the same transporters [7,353,354,355,356,357,358,359].

Accordingly, Choudhury et al. [212] found that the toxicity of As was reduced when plants of *Oryza sativa* were supplied with a Pi source. Due to the presence of plant-available Pi in the rhizosphere, the uptake of As by plant roots was greatly reduced, leading to an enhancement in plant growth, proteins, and carbohydrate and antioxidant enzymes activities [360]. Pigna et al. [361] showed that arsenate reductase enzyme was upregulated in wheat in the presence of Pi. This enzyme plays a pivotal role in As reduction and its subsequent sequestration in vacuoles in the form of an As(III)–PC complex. This type of sequestration is an important mechanism of As tolerance.

**Figure 6:**
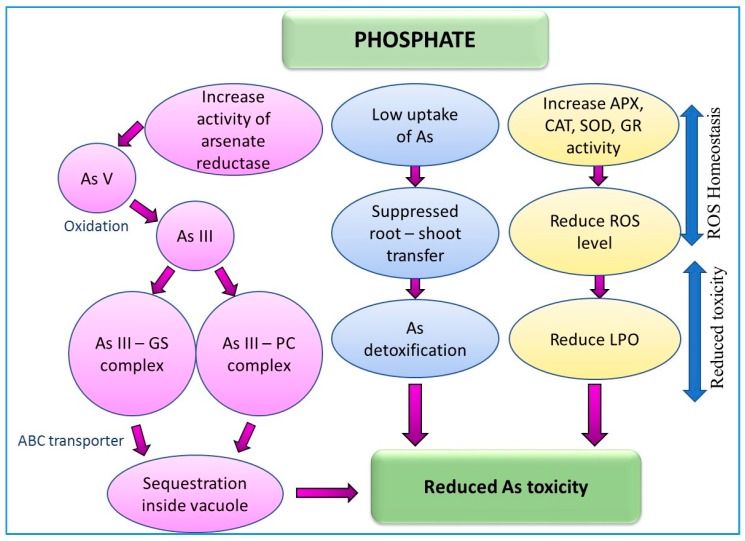
Impact of phosphate in plants under arsenic (As) stress. Phosphate provides tolerance against As by reducing arsenate (As(V)) uptake and limiting its translocation to shoots, arsenate reductase-mediated conversion of arsenate to arsenite (As(III)), and its subsequent sequestration into vacuoles, scavenges reactive oxygen species (ROS), and reduces oxidative stress by increasing the activities of antioxidant enzymes.

Niazi et al. [7] concluded that plant biomass, pigment concentrations and gas exchange attributes of *Brassica napus* plants, under As stress, were significantly improved with the addition of Pi to growth medium. They observed that As uptake and its root–shoot translocation was enhanced, indicating the importance of Pi supply for As phytoremediation. *Pteris vittata* has been extensively studied for its role in phytoremediation of As-contaminated soils [359,362,363,364,365,366]. The As-hyperaccumulator, *P. vittata*, is very efficient in the phytoextraction of As as it can hyperaccumulate As through high-affinity Pi transporters and reduce it to As(III), ultimately sequestering into frond vacuoles [363,365] (Figure 6).

It has been reported that As(V) adversely affects Pi metabolism and prevents secretion of various root exudates from plants [151,367]. Nevertheless, in the case of *Pteris vittata*, which is tolerant and an As-hyperaccumulator, more exudates were secreted under As supply in growth medium [151,368]. Fu et al. [362] revealed that *Pteris vittata* was more efficient than *Pteris ensiformis* in Pi acquisition from insoluble phosphate rock; the former species secreted more phytic acid in root exudates, which solubilized more phosphate rock and resultantly accumulated more As.

Karimi et al. [369] studied the interaction of As and Pi in *Isatis cappadocica* under hydroponic conditions. They confirmed that P supplementation significantly reduced As accumulation, its root to shoot transport and hence reduced As toxicity in this plant species. It has also been reported that Pi protects plants against oxidative damage upon their exposure to As [128,161]. Gomes et al. [128] noticed that, due to Pi addition, activities of antioxidant enzymes such as CAT and APX were significantly increased and scavenged ROS in plants growing under As stress.

Souri et al. [44] found that the combined application of high doses (1600 μΜ) of Pi compared with the lower dose (5 μΜ), was more effective in reducing As accumulation and lipid peroxidation in *Isatis* *cappadocica*. Moreover, Pi supplementation enhanced GR activity by regulating glutathione biosynthesis. High soil Pi level had an ameliorating effect on soybean growing under the combined stress of fluoride and As [54]. The protective effect of Pi was exhibited by an increase in chlorophyll concentrations and more protection against oxidative damage [54,361] (Figure 6).

## 10. Conclusions

It is evident from the above thorough and critical discussion that As exposure adversely affects plants at biochemical and molecular levels, and influences a majority of physiological responses, such as inhibition in overall growth processes, photosynthetic efficiency, and biomass accumulation. Arsenic can induce oxidative stress via the enhanced production and/or inefficient elimination of ROS and consequently damage lipids, proteins, and nucleic acids, and interferes with various metabolic pathways, either directly, as competitive inhibitors of Pi, or indirectly, by interfering with activities of certain key enzymes.

Additionally, As is known to reduce and/or inhibit the process of seed germination, root/shoot growth, and various earlier developmental processes, that occurs during initial stages of seedling development. Plants have evolved several mechanisms to overcome the toxic effects of As, such as compartmentalization, synthesis of As binding proteins (PCs and metallothioneins), and/or accumulation of compatible solutes such as proline, glycine betaine, and mannitol.

The oxidative stress is mitigated by the higher activities of antioxidant enzymes such as SOD, POD, CAT, APX, and GR. Furthermore, exogenous application of proline, NO, SA, and P are largely reported to provide tolerance against As through various mechanisms.

Although partially understood, future research is warranted to unravel the toxic effects of As on lipid, protein, and DNA metabolism during germination and post-germination phases of plant development. Moreover, the role of exogenous application of proline, NO, SA, and Pi has been mostly investigated under hydroponic conditions—therefore, further studies are needed to examine these aspects in different types of soil. 

Toxicity and defense mechanisms can be precisely determined by answering some important questions:

(i) How does As affect the germination and post-germination phases of plant development at the biochemical and molecular level?
(ii) What are the deleterious consequences (at the gene level) of As toxicity to plants and its organs?
(iii) How can plant toxicity symptoms be minimized without inducing any permanent damage to the plants?
(iv) How and to what extent can the exogenous application of various agents protect plants against As stress under soil conditions?

## Figures and Tables

**Table 1:** Arsenic induced physiological and biochemical changes in plants.

Plant Species	Growth Medium	As(V/III) Concentration	Effects	References
*Cicer arietinum* L.	Soil	As(V) (0, 20 mg kg^−1^)	Reduction in essential and non-essential amino acids and Fe concentrations. Over expression of dehydration responsive genes (MIPS, PGIP, and DRE). Reduction in antioxidant enzyme activities (GR, CAT, SOD, APX, and GPX).	[45]
*Zea mays* L.	Soil	As(III) (0, 150 μM)	Reduction in gas exchange attributes (photosynthetic rate, transpiration rate, stomatal conductance) and chlorophyll concentrations.	[33]
*Zea mays* L.	Soil	As(V) (0, 40, 80, 120 mg kg^−1^),	Increase in shoot As and P concentrations, reduction in pigment concentrations (chlorophyll A, chlorophyll B, and total chlorophyll) and gas exchange attributes.	[8]
*Brassica napus* and *Brassica juncea*	Soil	As(V) (0, 25, 50 and 75 mg As kg^−1^)	Reduction in growth attributes (leaf area, plant height, number of leaves, shoot and root dry biomass), gas exchange attributes (photosynthetic rate, transpiration rate, stomatal conductance), photosynthetic pigments and water use efficiency.	[7]
*Vigna mungo* L.	Soil	As(V) (0, 100 and 200 µM)	Chlorophyll a, Chlorophyll b, total chlorophyll, and carotenoids decreased with increasing As concentration. Lipid peroxidation was increased. The activities of antioxidative enzymes (SOD, POD, and APX) except CAT were increased.	[34]
*Glycine max*	Soil	As(V) and As(III) (0, 25 µM)	Changes in the expression of a key messenger (Phosphatidic acid) via phospholipase D and phospholipase C. Moreover, a rapid and significant stomatal closure.	[43]
*Glycine max*	Soil	As(V) and As(III) (0, 25, 50, 100 and 200 µM)	Reduction in chlorophyll content and increase in lipid peroxidation. Reduction in root cortex area, broken cells in the outer cortical layer and cell death of root tips. Dark deposits in cortex cells and within phloem cell walls and xylem vessel elements.	[21]
*Oryza sativa* L.	Hydroponic	As(V) (0, 50 µM)	Increased leakage of electrolytes and increased root arsenate reductase activity along with relatively lower root to shoot As translocation in As tolerant rice genotype BRRI 33 than in sensitive genotype BRRI 51. Decrease in Pi content and increase in PCs content in roots.	[32]
*Boehmeria nivea* L.	Hydroponic	As(III) (0, 5, 10, 15, and 20 mg L^−1^)	Reduction in chlorophyll concentrations, relative water concentrations, SOD and CAT activities. Increase in H_2_O_2_, malondialdehyde (MDA) content, and electrolyte leakage.	[163]
*Oryza sativa* L.	Hydroponic	As(III) (0, 25 mM)	Carbohydrate metabolism and photosynthesis were greatly affected, however, As did not caused any significant oxidative damage to plants.	[164]
*Oryza sativa* L. var. Triguna	Hydroponic	As(III) (0, 50 µM)	Reduction in shoot and root growth, biomass production, and protein concentrations.	[165]
Aquatic plants species (*Vallisneria gigantea, Azolla filiculoides* and *Lemna minor*	Hydroponic	As(V) 2 ppm	Changes in fluorescence spectra and damage to photosystem II.	[166]
*Brassica juncea* L.	Soil	As(V) (0.0, 0.1, 0.2, and 0.3 mM)	Affected plant growth and biochemical stress indicators such as protein content, lipid peroxidation, and antioxidative enzymes (SOD, CAT, POD, APX, GR).	[167]
*Pisum sativum* L.	Hydroponic containing NaHS (0, 100 µM)	As(V) (0, 50 µM)	As uptake caused reduction in chlorophyll fluorescence, nitrogen content concentrations of H_2_S and nitric oxide (NO). The activities of cysteine desulfhydrase and nitrate reductase were also decreased. Increasing levels of ROS caused damage to lipids, proteins, and membranes.	[168]
*Oryza sativa* L.	Hydroponic	As(V) (0, 100 µM)	Increases in hydrogen peroxide and lipid peroxidation.	[24]
*Anadenanthera peregrine, Myracrodruon urundeuva*	Soil	As(V) (0, 10, 50, and 100 mg L^−1^)	Increase in hydrogen peroxide and lipid peroxidation.	[128]
*Trigonella foe num_graecum* L.	Soil	As(V) (0, 10, 20, and 30 mg As kg^−1^)	Reductions in radicle length, dry weight, and chlorophyll content.	[37]
*Hydrilla verticillata*	Hydroponic	As(V) (0, 100, and 500 µΜ)	Decline in chlorophyll content and rate of photosynthesis.	[169]
*Oryza sativa* L.	Hydroponic	As(III) (0, 50, 150, and 300 μM)	Reductions in seed germination; root and shoot length; chlorophyll and protein content, and genomic stability.	[170]
*Oryza sativa* L.	Soil culture (field study)	Groundwater As concentrations (17, 27, and 53 μg L^−1^) and soil As concentrations (10.4, 12.6, and 15.5 μg g^−1^)	Both essential and non-essential amino acids were decreased as the grain As concentration was increased in high As accumulating rice genotypes. Non-essential amino acids were increased in low As accumulating rice genotypes.	[171]
*Vigna mungo*	Soil	As(V) (0, 2.8 mM)	Delayed nodule formation and reduction in nitrogenase activity.	[172]
*Cicer arietinum* L.	Soil	As(V) (0, 5 mg kg^−1^)	Reduction in chlorophyll, relative leaf water, sucrose, proteins, starch, and sugars concentrations. Reduction in Ca, P, Fe, and amino acids like; Lys, Met, Pro, Thr, Trp, and Val.	[159]
*Lemna minor* L.	Hydroponic	As(V) and As(III) (0, 1, 4, 16, and 64 µM)	Reduction in chlorophyll, and increase in electrolyte leakage and lipid peroxidation.	[162]
*Helianthus annuus*	Soil	As(V) (0, 30, and 60 mg kg^−1^)	Reductions in plant growth and ionic concentrations (K, Ca, Mg, Si, Fe, Zn, Cu, Rb, and Sr).	[161]
*Festuca arundinacea*	Hydroponic	As(V) (0, 25 mΜ)	Excessive ROS accumulation, membrane perturbation and lipid peroxidation.	[173]
*Cicer arietinum* L.	Soil	As(V) (0, 30, and 60 mg kg^−1^)	Increase in H_2_O_2_ content and lipid peroxidation. Reduction in SOD and non-enzymatic antioxidants activities. Increase in CAT and APX activities.	[174]
*Trifolium pretense*	Soil	As(V) (0, 5, 10, and 50 mg kg^−1^)	Increase in SOD, POD, and glutathione activities. Reduction in chlorophyll and carotenoid concentrations.	[175]

**Table 2:** Arsenic detoxification mechanisms in plants.

Plant Species	Growth Medium	As(V/III) Concentration	Mechanisms/Effects	References
*Glycine max* L.	Soil with two P levels (21 and 8 mg P kg^−1^)	As(V) (0, 10, 50 and 100 mg As kg^−1^) with different levels of fluoride (F)	As caused more oxidative damage in low P soil than in high P soil. High soil P mitigated oxidative stress by higher increase in antioxidant activities (SOD, CAT, POX, and glutathione) and an increase in chlorophyll concentrations.	[54]
*Brassica juncea cultivars*; *Varuna* and *Pusa Jagannath (PJn)*	Hydroponic	As(III) (0, 50, 150, 300 µM)	Sulfur concentrations, thiol-related proteins, and phytochemicals played their protective role against oxidative stress. The higher levels of total and aliphatic glucosinolate (GSL) were responsible for higher As tolerance in in Varuna than PJn.	[25]
Brassica species	Soil with P levels (0, 50 and 100 (mg kg^−1^)	As(V) (0, 25, 50, 75 mg kg^−1^)	Pi application under As stress improved plant growth, photosynthetic pigments, gas exchange attributes (photosynthetic rate, transpiration rate, stomatal conductance), and water use efficiency.	[7]
*Helianthus annuus* L.	Hydroponic with SA concentrations (0, 10, 50, and 100 μM)	As(V) (0, 10 µΜ)	SA application mitigated the adverse effects of As on plant growth by reducing the oxidative stress and increasing the activities of (CAT), (APX), and (GPX), whereas the activities of (SOD) and (POD) were decreased.	[52]
*Oryza sativa* L.	Hydroponic with SNP (0.0 and 30 μM) as NO donor	As(III) (0.0, 25 µΜ)	SNP supply caused a reduction in As accumulation, ROS production and cell death. NO reduced As toxicity by modulating metal transporters (NIP, NRAMP, ABC, and iron transporters), stress-related genes, and secondary metabolism genes, signaling, amino acid and hormones such as jasmonic acid concentrations.	[39]
*Oryza sativa* L.	Hydroponic with salicylic acid (SA; 40 µM) and nitric oxide (NO as SNP; 30 µM)	As(III) (0, 25 µM)	Exogenous supply of SA lessened As(III)induced oxidative stress by increasing the activities of antioxidant enzymes, particularly SOD, CAT, and APX. SA and NO both restricted the accumulation of As in shoots possibly by downregulating *OsLsi2* gene. Nitric oxide mitigated As(III)induced chlorosis by increasing shoot Fe uptake.	[26]
*Pteris vittata* and *Vetiveria zizanioides*	Hydroponic	As(V) (0, 10, 20, 30, and 50 mg L^−1^)	*Pteris vittata* accumulated more As in fronds and showed higher activities of antioxidant enzymes (SOD, APX, CAT, and GPX) in fronds. *Vetiveria zizanioides* accumulated more As in roots and showed higher activities of antioxidant enzymes in roots.	[273]
*Glycine max*	Soil	As(V) and As(III) (0, 25, 50, 100, and 200 µM)	Increased activities of antioxidant enzymes such as total peroxidases (Px) and superoxide dismutase (SOD) both in shoot and root.	[43]
*Oryza sativa* L.	Hydroponic	As(V) (0, 50 µM)	Increased root arsenate reductase activity and PCs content in roots resulting in relatively lower root to shoot As translocation. Increased activities of antioxidants (CAT, POD, SOD, GR) and an increase in concentrations of amino acids (glutathione, cysteine methionine, and proline) in As tolerant rice genotype.	[32]
*Oryza sativa* L.	Hydroponic with three S levels (0.5, 3.5 and 5.0 mM)	As(V) (50 µM)	Exogenous supply of sulfur (S) increased As accumulation in roots and decreased its transport to shoot by reducing the expression of potent transporters (*OsLsi*1 and *OsLsi*2). The activities of antioxidant enzymes were increased and the synthesis of PCs was increased which caused As complexation in the roots.	[51]
As-hyperaccumulator *Pteris vittata*	MS agar medium containing arsenic resistant bacteria	As(V) (37.5 mg kg^−1^)	Reduced As induced toxicity by efficient As III efflux into external environment and As III translocation to the fronds.	[298]
*Arabidopsis thaliana*	Hydroponic	As(III) (5 mg/L) and As(V) (10 mg/L)	Reduction of As(V) to As(III)by the effect of root excreted organic acids and efflux of As(III)from plant roots after in vivo reduction of As(V) to As(III).	[299]
*Oryza sativa* L.	Hydroponic with 0 and 2 mM Si	As(III) (0 and 25 mM)	Application of Silicon (Si) reduced As uptake by plants and improved photosynthetic attributes by changing the expression of genes involved in As uptake and translocation.	[164]
*Oryza sativa* L. var. Triguna	Hydroponic inoculated with alga; *Chlorella vulgaris* and *Nannochlropsis* sp.	As(III) (0, 50 µM)	Algal inoculum reduced As toxicity and improved plant As tolerance by reducing As uptake and modulating the activities of antioxidant enzymes.	[165]
*Nicotiana tabacum* L.	Hydroponic inoculated with Endomycorrhizal fungus *Funneliformis mosseae*	As(V) (0, 1 and 30 µM)	Endomycorrhizal fungus *Funneliformis mosseae* increased the concentrations of PCs and antioxidant glutathione (GSH) and reduced the uptake of As and Cd in roots and leaves.	[50]
*Oryza sativa* L.	Hydroponic with sulfur (0, 0.5, 3.5, 5.0 mM)	As(III) 0, 25 µM), and As(V) (0, 50µM)	Exogenous application of S particularly the highest level, restricted As in roots due to its complexation with non-protein thiols and PCs. Oxidative stress was mitigated by limited generation of hydrogen peroxide and higher activities of antioxidant enzyme.	[300]
*Brassica juncea* L.	Hydroponic	As(V) (0.0, 0.1, 0.2, and 0.3 mM)	Synthesis of brassinosteroids (castasterone, teasterone, 24-epibrassinolide, and ty-phasterol) and overexpression of antioxidant enzymes.	[167]
*Oryza sativa* L.	Hydroponic with Se (0, 20 µM) and auxin (0, 3 µM)	As(III) (0, 150 µM)	Co-application of selenium (Se) and auxin to rice seedlings reduced As toxicity by increasing growth, chlorophyll, protein cysteine and proline concentrations and decreasing MDA level in the cell.	[47]
*Lettuce sativa* L.	Hydroponic with 100 μM sodium nitroprusside (SNP)	As(V) (0, (50 µM)	Exogenous supply of NO in the form of SPN reduced root to shoot translocation of As and decreased the oxidative damage by decreasing the concentrations of H_2_O_2_ and MDA.	[272]
*Pisum sativum* L.	Hydroponic containing NaHS (0, 100 µM)	As(V) 0, (50 µM)	Addition of hydrogen sulfide to growth medium improved plant As tolerance by increasing the concentrations of H_2_S and NO and reducing the oxidative damage caused by ROS. Arsenic accumulation was decreased and AsA–GSH cycle was upregulated to offset ROS-mediated damage to cell.	[168]
*Solanum melongena* L.	Hydroponic with 25 µM proline	As(V) (0, 5, and 25 µM)	Exogenous application of proline reduced As accumulation. Deleterious effects of As on photosystem-II (PSII) were ameliorated, and chlorophyll concentrations were improved. Oxidative stress was mitigated by the higher activities of antioxidant enzymes (SOD, POD, CAT, and glutathione-*S*-transferase; GST). The activity of proline biosynthesis enzyme; Δ1-pyrroline-5-carboxylatesynthetase was upregulated, and proline dehydrogenase was downregulated.	[277]
*Oryza sativa* L.	Hydroponic culture with Si (0, 2 mM)	As(III) (0, 25 μM)	Silicon (Si) application decreased As accumulation in leaves and improved photosynthetic performance (net CO_2_ assimilation rate, stomatal conductance, and mesophyll conductance) of rice plants in a genotype and time-dependent manner.	[164]
*Cicer aritenum* L.	Soil with bacterial inoculation (*Acinetobacter* sp)	As(V) (0, 10 mg kg^−1^)	Bacterial inoculation significantly increased root and shoot biomass, total chlorophyll protein and carotenoid concentrations and decreased As uptake and electrolyte leakage by reducing MDA concentrations.	[271]
*Oryza sativa* L.	Hydroponic	As(III) (0, 10, and 25 µM) and As(V) (0, 10, and 50 µM)	Higher activities of antioxidant enzymes such as superoxide dismutase (SOD), ascorbate peroxidase (APX), and guiacol peroxidase (GPX), and higher concentrations of stress responsive amino acids (glycine, cysteine, proline, glutamic acid) in high As accumulating genotype than low As accumulating genotypes.	[289]
*Triticum aestivum* L.	Hydroponic with 0 and 0.25 mM SNP	As(V) (0, 0.25, and 0.5 mM)	Exogenous application of nitric oxide (NO) in the form of sodium nitroprusside, (SNP) increased the RWC, chlorophyll and proline concentrations, AsA and GSH, glyoxalase I and glyoxalase II concentrations, and the activities of antioxidants (CAT, GPX, GR, dehydroascorbate reductase (DHAR).	[301]
